# Multiscale Mechanobiology in Brain Physiology and Diseases

**DOI:** 10.3389/fcell.2022.823857

**Published:** 2022-03-28

**Authors:** Anthony Procès, Marine Luciano, Yohalie Kalukula, Laurence Ris, Sylvain Gabriele

**Affiliations:** ^1^ Mechanobiology and Biomaterials group, Interfaces and Complex Fluids Laboratory, Research Institute for Biosciences, University of Mons, Mons, Belgium; ^2^ Neurosciences Department, Research Institute for Biosciences, University of Mons, Mons, Belgium

**Keywords:** mechanobiology, brain cells, brain tissues, mechanotransduction, brain diseases, extracellular matrix, cytoskeleton

## Abstract

Increasing evidence suggests that mechanics play a critical role in regulating brain function at different scales. Downstream integration of mechanical inputs into biochemical signals and genomic pathways causes observable and measurable effects on brain cell fate and can also lead to important pathological consequences. Despite recent advances, the mechanical forces that influence neuronal processes remain largely unexplored, and how endogenous mechanical forces are detected and transduced by brain cells into biochemical and genetic programs have received less attention. In this review, we described the composition of brain tissues and their pronounced microstructural heterogeneity. We discuss the individual role of neuronal and glial cell mechanics in brain homeostasis and diseases. We highlight how changes in the composition and mechanical properties of the extracellular matrix can modulate brain cell functions and describe key mechanisms of the mechanosensing process. We then consider the contribution of mechanobiology in the emergence of brain diseases by providing a critical review on traumatic brain injury, neurodegenerative diseases, and neuroblastoma. We show that a better understanding of the mechanobiology of brain tissues will require to manipulate the physico-chemical parameters of the cell microenvironment, and to develop three-dimensional models that can recapitulate the complexity and spatial diversity of brain tissues in a reproducible and predictable manner. Collectively, these emerging insights shed new light on the importance of mechanobiology and its implication in brain and nerve diseases.

## Introduction

**FIGURE 1:**
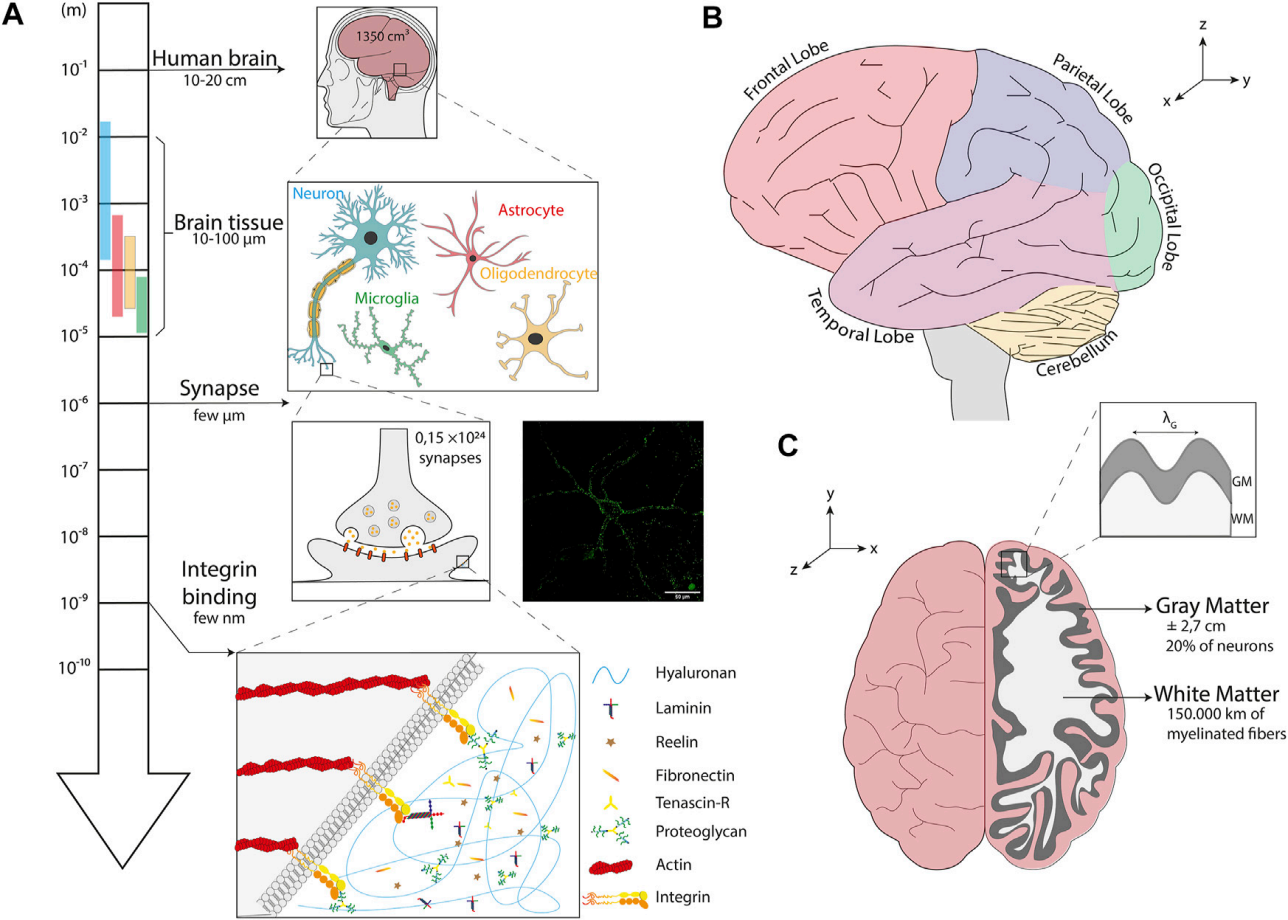
The multi-scale structure of the human brain. **(A)** Homeostasis of brain tissues mainly relies on the interaction between four cell types: neurons, microglial cells, astrocytes and oligodendrocytes. At the subcellular scale, synapses allow the transmission of an electrical or chemical signal from a neuron to another one or to the target effector cell. The inset shows an image of a neuron isolated from an organotypic slice of hippocampus labeled with synaptotagmin (in green), a presynaptic calcium sensor. The scale bar is 50 µm. At the molecular scale, transmembrane integrins connect proteins of the extracellular matrix to cytoskeletal components. **(B)** Illustration of a sagittal section of a normal human brain whose different lobes are color-coded: the frontal lobe in red, the parietal lobe in blue, the occipital lobe in green, the temporal lobe in purple and the cerebellum in yellow. **(C)** Illustration of a top view of a normal human brain with a cross section of the right lobe showing the organization of the cerebral cortex (gray matter) and subcortical regions (white matter). The inset depicts the wavelength (λ_G_) of cortical folds, the grey matter (GM) and the white matter (WM).

It is now well accepted that brain tissues are one of the most complex and compliant tissue in the human body (Budday et al., 2020). While neuroscience has mostly been limited to electrophysiological, biochemical, and genetic studies over the past few decades, emerging evidence confirms that mechanobiology plays a critical role in modulating brain function and dysfunction (Tyler, 2012). Indeed, accumulative works suggest that mechanical properties of the cell microenvironment can control developmental processes (Koser et al., 2016) and are involved in the progression of brain diseases (Ngo and Harley, 2021), while external mechanical forces can lead to brain injury (Meaney and Smith, 2011). Consequently, a better understanding of mechanobiological processes in brain tissues requires sophisticated *in vitro* models that capture more realistically the complex characteristics of brain tissues through engineered multi-scale platforms. These novel platforms must allow access to the molecular level, where transduction of mechanical signals occurs, to the cellular level, where mechanotransduction processes take place, and to inter-cellular interactions that control cross-talks between different brain cell types (Figure 1A).

Natural mechanical stimuli are part of the life of each individual and do not inevitably lead to brain injury. Indeed, the human nervous system is well organized and protected in many ways. It is divided into the central nervous system (CNS), which includes the brain and spinal cord, and the peripheric nervous system composed of the nerve, which connects the brain and spinal cord to tissues and organs (Barker et al., 2019). The first is enclosed by the skull, which provides an important physical barrier to all external aggressions and is also coated by three layers of membranes known as the meninges (dura mater, arachnoid mater, and pia mater), which protect the brain and spinal cord. The CNS is irrigated by the cerebrospinal fluid, which also assumes a role in the protection of neuronal tissue, both from an immunological and mechanical point of view (Saunders et al., 2016). The blood-brain barrier restricts the passage of pathogens, the diffusion of solutes in the blood, and large or hydrophilic molecules into the cerebrospinal fluid, while allowing the diffusion of hydrophobic molecules and small non-polar molecules (Daneman and Prat, 2015).

The high level of protection of the CNS allows to ensure the development is properly conducted through a precise orchestration sequence of genetic, biochemical, and physical events. For instance, rapid movements of the head or torsion of the backbone can be absorbed by the physical barriers, whereas inertia load is partially resorbed by the cerebrospinal fluid irrigating the meninges. Physical forces play a central role in translating the molecular and cellular mechanisms during neurodevelopment and maturation of the nervous system (Budday et al., 2015b). In some cases, the load can be above the limit leading to damaged cells and tissues (Bilston, 2011).

This review aims to describe the basic physical principles underlying brain function and to present our current understanding of the role of mechanical forces in physiological and pathological situations. We ambition to motivate further research in the field of brain and nerve mechanobiology by highlighting the need for a global approach to brain mechanics at a multiscale level.

## Scale of the Human Brain

Understanding how brain tissue mechanobiology is intimately linked with neurophysiology and brain disease progression requires to take the specificity and complexity of the human brain into account. Using a large sample of human brains from the general population, it has been established that the mean human brain weighs about 1,375 g for an average volume of ∼1,350 cm^3^, resulting in a volume mass of ∼1,019 kg/m³, which is comparable to calcium oxide. The total brain surface area is ∼1,820 cm^2^ and the average cortical thickness was found to be ∼2.7 mm (Pakkenberg and Gundersen, 1997). Interestingly, about 20% of neurons are located in the cerebral cortex (Herculano-Houzel, 2009) and each cortical neuron forms an average of ∼7,000 synaptic connections with other neurons, resulting in a total of 0.15 quadrillion synapses (Pakkenberg, 2003). These numbers allow to estimate that more than 150,000 kms of myelinated fibers browse the human brain, which is approximately the average distance covered by a car during its entire lifetime (Figures 1A–C).

Although the human brain makes up only ∼2% of the body weight, it is well established that the human brain uses more energy than any other organ, accounting for up to 20% of the body’s baseline energy (Shokri-Kojori et al., 2019). Until recently, it was widely accepted that this energy was mainly used to fuel electrical impulses that neurons use to communicate with neighbors. This concept was refined by using magnetic resonance spectroscopy (MRS) to measure the brain energy production during activity shifts. It was found that two-thirds of the brain’s energy budget is used to support the firing of nerve cells, whereas the remaining third refers to cell-health maintenance (Raichle and Gusnard, 2002). This theory has been very recently confirmed by showing that synaptic vesicle (SV) pools are a major source of presynaptic basal energy consumption. Indeed, it was found that basal metabolic processes arise from SV-resident V-ATPases compensating for a hidden resting H+ efflux from the SV lumen, whereas that this steady-state H+ efflux is mediated by vesicular neurotransmitter transporters, is independent of the SV cycle, accounts for up to 44% of the resting synaptic energy consumption, and contributes substantially to nerve terminal intolerance of fuel deprivation (Pulido and Ryan, 2021).

It is usually reported in textbooks, reviews, and even original articles that the human brain is composed of about 100 billion neurons and about 10 more glial cells, even though no clear references are cited (Herculano-Houzel, 2009). The lack of original references for these numbers may lead the reader to believe that the cellular composition of the human brain has long been determined and can be used for developing new bioengineered platforms. Even if a direct estimation of the number of different cell types in the entire human brain is difficult to obtain, the relative abundance of each cell type in different parts of the brain is considered as a determinant of neural function and behavior (Williams and Herrup, 1988; Herculano-Houzel, 2011). However, determining glial cell counts is particularly challenging due to their small size and the difficulty to isolate them (von Bartheld et al., 2016).

To address this challenge, the isotropic fractionation method was introduced to transform an intact brain into a soup of nuclei (Gabi et al., 2016). Entire brains were sliced up into regions of interest (e.g., cerebellum, cerebral cortex, etc.), tissues were ground-up and then dissolved in saline detergent to harvest nuclei from both neurons and glia. An anti-NeuN antibody was used to bind specifically to proteins of neuronal nuclei and subtract that number from the total number of nuclei to determine the fraction of glial cells in each brain section. By using this method, it was found that the human brain contains about 170.68 billion cells, 86.1 billion of which are neurons and 84.6 billion of which are glial cells, thus debunking the myth there are at least 10 times as many glia as neurons in the human brain (Herculano-Houzel, 2005). Interestingly, it was also suggested that the ratio of glia to neurons differs significantly between different brain regions. In the cerebral cortex, 60.84 billion cells are glia, while only 16.34 billion cells are neurons, giving this large region glia to neuron ratio of about 3.72. An inversed situation was found in the cerebellum, that contains 69.03 billion neurons and only 16.04 glial cells, which means there are about 4.3 neurons for every glia in this region (Azevedo et al., 2009). The variations of the glia to neuron ratio is fascinating and it could be interesting to further investigate whether the difference of glia to neuron sub-populations can be involved in the modulation of the mechanical properties of brain tissue. In addition, it also highlights the importance of robust numbers for developing advanced engineering approaches to study the exact role of glial cells by considering their structural diversity, functional versatility, and the fact that they can change the behavior of firing neurons.

In this way, glial cells are extensively studied to understand whether they must be only considered as a “glue” for neurons or whether they also support them by providing a mechanical scaffold. To answer this question, the mechanobiology of brain tissues is intensively studied and many efforts focus on the determination of the individual mechanical characteristics of the main brain cell types.

## Mechanobiology of Brain Tissues

*In vitro* recapitulation of the native microenvironment of neurons and glial cells is crucial for studying cellular responses and creating biomaterials mimicking brain tissues. The brain is a complex tissue that is extremely soft and often considered as the softest tissue in the human body. Brain tissues are known to be incompressible (Brands et al., 2004) due to a large amounts of proteoglycans, which are heavily glycosylated proteins that bind water (Lau et al., 2013), leading to a relatively high amount of water of approximately 73–85% of the total mass. Interestingly, lipids account for roughly 60% of the total dry weight of the brain (O’Brien and Sampson, 1965; Chang et al., 2009).

**FIGURE 2:**
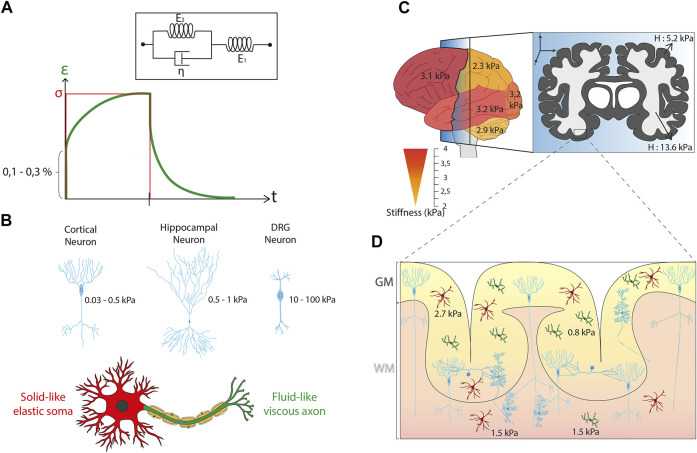
Mechanical properties of brain cells and tissues. **(A)** Kelvin-Voigt representation of the Standard Linear Solid (SLS) model that describes a viscoelastic material with two systems in series. The first system is called the Kelvin arm and is formed by a spring (E_2_) and a dashpot (η) in parallel. The other arm is only made of a spring (E_1_). These SLS model predicts the temporal evolution of the strain (ε, solid black curve) in response to the application of a constant stress (σ) depicted in red line. **(B)** Sagittal view of the normal human brain color-coded with a gradient of stiffness starting from yellow (∼2 kPa) to red (∼4 kPa) that illustrates the differences of mechanical properties in brain tissues according to the different regions of interest. Parietal lobe and cerebellum are softer than frontal, parietal, and occipital lobes. The frontal section illustrates the anatomical distribution of white (∼13.6 kPa) and grey (∼5.2 kPa) matter. **(C)** Illustration of the mean stiffness observed in different neuron types. A cortical neuron exhibits a typical stiffness between 0.03 and 0.5 kPa, a neuron from the hippocampus shows a stiffness of 0.5–1 kPa, and a DRG neuron is characterized by a mean stiffness of 10–100 kPa. The mechanical compartmentalization of a neuron is presented with a solid-like elastic Soma (in red) and a fluid-like viscous axon (in green). **(D)** Cross-section of the cerebral cortex indicating the individual mechanical properties of the glial cells as well as their spatial differences with values of ∼0.85 kPa for cortical microglia cells, ∼1.5 kPa for subcortical microglia cells, ∼2.7 kPa for cortical astrocytes and ∼1.5 kPa for subcortical astrocytes. It is interesting to note the non-homogeneous distribution of glial cells in brain tissues with a larger cell density in grey matter.

This very specific composition of brain tissues leads to elaborated rheological properties. Although mechanical properties of brain tissues are difficult to test experimentally and to model theoretically (Goriely et al., 2015), it is now well accepted that brain stiffness increases with age, starting at the developing brain (Antonovaite et al., 2021). As a matter of fact, brain tissue acts as a linear viscoelastic material under very small strain in order of ∼0.1–0.3% (Bilston et al., 1997; Nicolle et al., 2005) (See Box 1 and Figure 2A). Interestingly, storage (i.e. the elastic part) and loss (i.e. the viscous part) moduli of brain tissues both increase with frequency (Bilston et al., 1997). When the strain goes beyond the limit of linear viscoelastic strain, brain tissues behave in a complex non-linear mode, with an apparent stiffness depending on both the strain and the type of loading. Low mechanical inputs induce reversible deformations, which are characteristic of elastic behavior. Under high deformations, brain tissues exhibit a plastic deformation as observed in non-linear viscoelastic materials (Budday et al., 2017). Moreover, brain tissues are unable to adapt over short time scales and can be therefore subject to damage and injury (Mc Intosh et al., 1998).

BOX 1 **Viscoelastic properties**

Viscoelasticity is a time-dependent mechanical property of synthetic and living materials that exhibit both viscous and elastic characteristics when undergoing deformation. Viscoelastic properties are usually evaluated by recording over time the degree of deformation of an object upon constant force application (i.e., creep) and then its recovery (i.e., relaxation). Purely elastic materials (Hookean solids) are described with a spring model that relates linearly stress (*σ*) to strain (*ɛ*) by the elastic modulus (E in N/m^2^ or Pa), such as E = σ*/ɛ*. The stress (*σ,* in Pa) is defined as the exerted force (in Newtons) per unit area (in m^2^), while the strain (ε, adimensional) is defined as changes in length with respect to initial length. The response of purely elastic materials to a creep-relaxation test is to undergo an instantaneous elastic strain upon loading, to maintain that strain as long as the load is applied, and then to undergo an instantaneous recovery upon removal of the load. Purely viscous fluids (Newtonian fluids) are described with a dashpot model that represents a piston-cylinder filled with a viscous fluid (*η* expressed in Pa.s). The dashpot responds with a strain-rate proportional to stress. Viscoelastic materials exhibit mechanical characteristics of both solids and fluids: at short time scales, they deform elastically, while they behave as viscous fluids at long time scales. Basically, viscoelastic materials can therefore be described as composite structures containing an elastic spring connected to a viscous dashpot, either in series (Maxwell configuration) or in parallel (Kelvin-Voigt configuration). By using more complex combinations of springs and dashpots, many different viscoelastic models were developed, such as the standard linear solid model (SLSM), to determine the viscoelastic properties of materials based on creep-relaxation experiments. Here we show the Kelvin form of the SLSM model (Figure 2A) that consists of two systems in series: the first contains a spring (*E*_*2*_) and a dashpot (*η*) in parallel and the second contains only a spring (*E*_*1*_). Upon loading the right-hand spring (*E*_*1*_) stretches immediately. The dashpot (*η*) then takes up the stress, transferring the load to the second spring (E_2_) as it slowly opens over time. Upon unloading the right-hand spring (*E*_*1*_) contracts immediately and then the left-hand spring (*E*_*2*_) slowly contracts, being held back by the dashpot. As shown in Figure 2A, the Kelvin form of the SLSM model allows to describe more realistically the viscoelastic responses of cells and tissues than simplest viscoelastic models (Wang and Kuhl, 2020).

Brain rheological properties are a key ingredient of the cortical folding process, by which brain tissues undergo morphological changes in terms of waving. Indeed, the brain cortex starts to form convex gyri and concave sulci, whose depth and length increase during development, which starts at the third trimester of gestation in humans. Different models of cortical folding have been reported that rely on a combination of biological and mechanical properties of brain tissues: i) axonal tension-driven folding which assumes that tension driven by axons triggers the buckling of the cortical layer (Essen, 1997), ii) differential tangential growth, which explains buckling by the more rapid growth of the external layer of the cortical plate than the inner layer lying on the elastic core (Richman et al., 1975; Tallinen et al., 2016) and iii) viscoelastic instability in a model of stress-induced growth, which includes a growing core under the resulting stresses from outer layer expansion producing a stress-growth relationship between inner and outer layer (white and gray matter) (Bayly et al., 2013). Altogether, these models illustrate the importance of mechanobiology to further understand fundamental biological mechanisms such as cortical folding.

However, inconsistency in sample preparation, postmortem timing, and testing conditions in most of the previous experiments prevents a reliable mechanical characterization of brain tissues (Hrapko et al., 2008). Despite this technical difficulty, the mechanical characterization of specific part of brain tissues is considered as an important issue for understanding the individual properties of white and gray matter. White matter is mostly composed of bundled myelinated axons, whereas gray matter contains numerous cellular bodies and relatively few myelinated axons. Interestingly, this structural difference is directly correlated with changes in mechanical properties. Indeed, white matter is approximately 30% stiffer than grey matter, with a stiffness of ∼1.9 kPa for white matter and ∼1.4 kPa for gray matter for bovine tissues in compression mode (Budday et al., 2015a). Magnetic resonance elastography (MRE) on the human brain has shown a shear modulus of ∼13.6 kPa for white matter and ∼5.2 kPa for grey matter (Kruse et al., 2008), while another group (Green et al., 2008) showed more similar shear stiffness values for white (∼3.1 kPa) and gray (∼2.7 kPa) matter (Figure 2B). White matter have also higher regional variations of stiffnesses, is more viscous, and shows longer relaxation times than gray matter (Pervin and Chen, 2009; Budday et al., 2015a). Even in the mature brain, significant differences in brain tissue stiffness were found between different regions, including the cerebellum, medulla, cortex, and pons (MacManus et al., 2017). Using MRE, cerebellum (∼2.9 kPa) and parietal (∼2.4 kPa) lobes were found to be softer than frontal (∼3.2 kPa), occipital (∼3.2 kPa), or temporal (∼3.2 kPa) lobes (Murphy et al., 2013) (Figure 2B). Interestingly, a correlation has been issued between the local stiffness and the underlying morphological properties with regions of high nuclear densities that appear softer than those with lower nuclear densities. For instance, the stiffness of the CA3 stratum pyramidal (CA3-SP) (high nuclear density) was found to be softer (∼1.5 kPa) than the stratum radiatum (CA3-SR) (low nuclear density) of ∼3 kPa.

These differences in mechanical properties between brain regions result from complex combinations of different cell types, which are embedded in an extracellular matrix (ECM) whose composition varies depending on the brain zone (Lam et al., 2019). Determining the mechanical properties of each brain cell type and ECM is therefore crucial for understanding how mechanical forces acting on the brain could be involved in the brain functioning and the development of pathological situations.

## Mechanobiology of Brain Cells

Neurons conduct nerve impulses and for this reason they are considered as the central functional unit of the brain, while glial cells maintain the biochemical homeostasis and serve as physical support to neurons (Franze et al., 2013). We will present in this section the current mechanical picture of neuronal and glial cells and how both cell types adapt to physico-chemical changes (topography, stiffness, or composition) of their microenvironment.

### Mechanical Properties of Neurons

Neurons are highly specialized cells that are primarily responsible for transmitting information through chemical and electrical signaling, in both the central and peripheral nervous systems. Since there is a broad range of functions performed by different types of neurons, there is also a wide variety of morphologies. The typical morphology of neurons are composed of a cell body, also called the Soma, that contains the nucleus and two other compartments: the dendrites, which are fine and branched cell processes that receive synaptic input from other neurons, and one axon that can reach a length of several meters with presynaptic terminals (Franze and Guck, 2010). The structural and mechanical properties of neurons are essentially defined by the spatial organization of their cytoskeletal filaments, which governs growth and regeneration processes, including axonal extension and the generation of traction forces, but also interactions with the surrounding environment, such as the ECM, glial cells, or other neurons (Marinval and Chew, 2021).

Several rheological studies have been conducted on different neuronal cell types and from different species, resulting in a discrepancy in stiffness values. Atomic Force Microscopy (AFM) measurements of mouse hippocampal neurons reported a stiffness between 480 and 970 Pa for the Soma (Lu et al., 2006). Cortical neurons were found to be softer, with a typical stiffness ranging from 30 to 500 Pa (Bernick et al., 2011; Spedden et al., 2012a; Spedden et al., 2012b). Interestingly, recent investigations with optical tweezers confirmed a very low stiffness for cortical neurons with a Young’s modulus around 50 Pa (Ayala et al., 2016). Dorsal root ganglion (DRG) neurons, which are a cluster of neurons (a ganglion) in a dorsal root of a spinal nerve, were found to be stiffer with a mean stiffness ranging from 10 to 100 kPa (Mustata et al., 2010; Spedden et al., 2012a; Martin et al., 2013). As discussed previously, neurons are compartmentalized cells which can exhibit different sub-cellular mechanical properties. By combining protein micropatterns and magnetic tweezers, it was found that the cell body is soft with a solid-like and stress-stiffening response, whereas the neurite compartment is stiffer and viscous-like (Grevesse et al., 2015). The growth cone, which directs the migration of neurons and mediates the formation of synapses (Kalil and Dent, 2014), was reported to have a Young’s modulus ranging from ∼0.4 to ∼40 kPa (Martin et al., 2013) (Xiong et al., 2009) (Figure 2C).

An intriguing question concerns the influence of the mechanical properties of individual neuronal and non-neuronal cells on the stiffness of specific regions of the brain. To address this question, the spatiotemporal stiffness of the mouse embryonic cerebral cortex and isolated cells from the same brain region was probed with AFM (Iwashita et al., 2014). A gradual stiffening of specific layers of the embryonic brain was observed over time, whereas the rigidity of neuronal progenitor cells remained constant over time. In addition, the cortical plate showed an initial increase in stiffness until E18.5 (embryonic stage) where it started to decrease, whereas the neuronal population of this layer showed a constant increase in their stiffness according to the maturation of microtubules (Iwashita et al., 2014). Altogether these results demonstrated that the mechanical behavior of individual cells cannot explain the temporal evolution of the tissue stiffness. Other reports performed on the cortical plate of mice and ferrets confirmed that this observation and suggested that the density of the cell population could modulate the mechanical properties of brain tissues (Nagasaka et al., 2016). To get a step further in understanding the temporal behaviour of brain tissue mechanics, future studies will have to focus on the variation of cell density across brain tissues and consider the composition and the stiffness of the cell matrix.

### Mechanical Properties of Glial Cells

Glial cells participate to form the microenvironment of neurons by filling most of the interstitial space. They ensure the maintenance of homeostasis, produce myelin and play a role in supporting and protecting nervous tissues by providing nutrients and oxygen, eliminating dead cells, and fighting pathogens (Jäkel and Dimou, 2017). Glial cells find the origin of their name from the Greek word “γλια” meaning “glue” and they were long considered as the glue of brain tissues, acting as a paste between neurons. This theory was challenged by probing with AFM the viscoelastic properties of individual glial cells and neurons in the CNS (Lu et al., 2006). Both cell types were found to exhibit predominant elastic properties but very low elastic moduli (<1 kPa). Glial cells were twice softer than neurons, with an elastic modulus of 300–520 Pa for astrocytes and 480–970 Pa for neurons. Interestingly, similar results were obtained for Müller glial cells, which are a type of retinal glial cells (Lu et al., 2006). Very recently, the stiffness of microglial cells was reported to be even softer with an elastic modulus ranging from 40 to 100 Pa (Rheinlaender et al., 2020). Interestingly, microglia derived from gray matter are intrinsically softer (842 Pa) than microglia derived from white matter (1,429 Pa), suggesting that their mechanical properties depend on their spatial location (van Wageningen et al., 2021). This characteristic was also recently confirmed for astrocytes. Indeed, astrocytes from the white matter (∼1.5 kPa) were found to be approximatively 1.8 times softer than astrocytes derived from the gray matter (∼2.7 kPa) (Antonovaite et al., 2020) (Figure 2D).

Taken together, these findings indicate that neuronal cells are surrounded by softer glial cells, which are constantly subjected to physical forces. It was shown recently that Schwann cells (SCs), which have a dense vimentin network, have a great ability to resist mechanical deformation with nuclei that are hard to deform, suggesting that adults SCs can mechanically protect the neurons they encase (Rosso et al., 2019). To further confirm this hypothesis, future studies should investigate the mechanical properties of the nucleus of glial cells and focus on nucleoskeletal interactions to better understand their mechanosensing properties and identify the mechanotransduction pathways in glial cells, which in turn may have an impact on neuronal network activity.

### Cytoskeleton, Nucleus and Molecular Clutch in Brain Cells

Even if it has been demonstrated very recently that the cortical stiffness of microglial cells is independent of substrate mechanics (Rheinlaender et al., 2020), the reorganization of the cell cytoskeleton allows many cell types to adapt their stiffness to that of their surroundings (Doss et al., 2020). Adherent cells can sense their mechanical environment through focal adhesions, which connect the cytoskeletal filaments to the ECM via transmembrane integrins and act as a mechanosensor (see Box 2). In addition, cells can also use adherens junctions to adapt their mechanical properties to the intercellular stress and the spatial confinement (Mohammed et al., 2019) imposed by neighbouring cells (Takeichi, 2007). Interestingly, adherens junctions bridge neighboring cells and the actin-myosin cytoskeleton, thereby contributing to mechanical coupling between cells (Indra et al., 2020). Adherent cells must adapt their shape and their mechanical properties in response to the physico-chemical properties of the ECM to perform their tasks, such as migration or differentiation. Much effort has gone into identify mechanotransduction pathways and the molecular process used by brain cells to convert mechanical stimuli into biochemical signals.

BOX 2 **Cytoskeleton of neuronal cells: structure and function**

**FIGURE 3:**
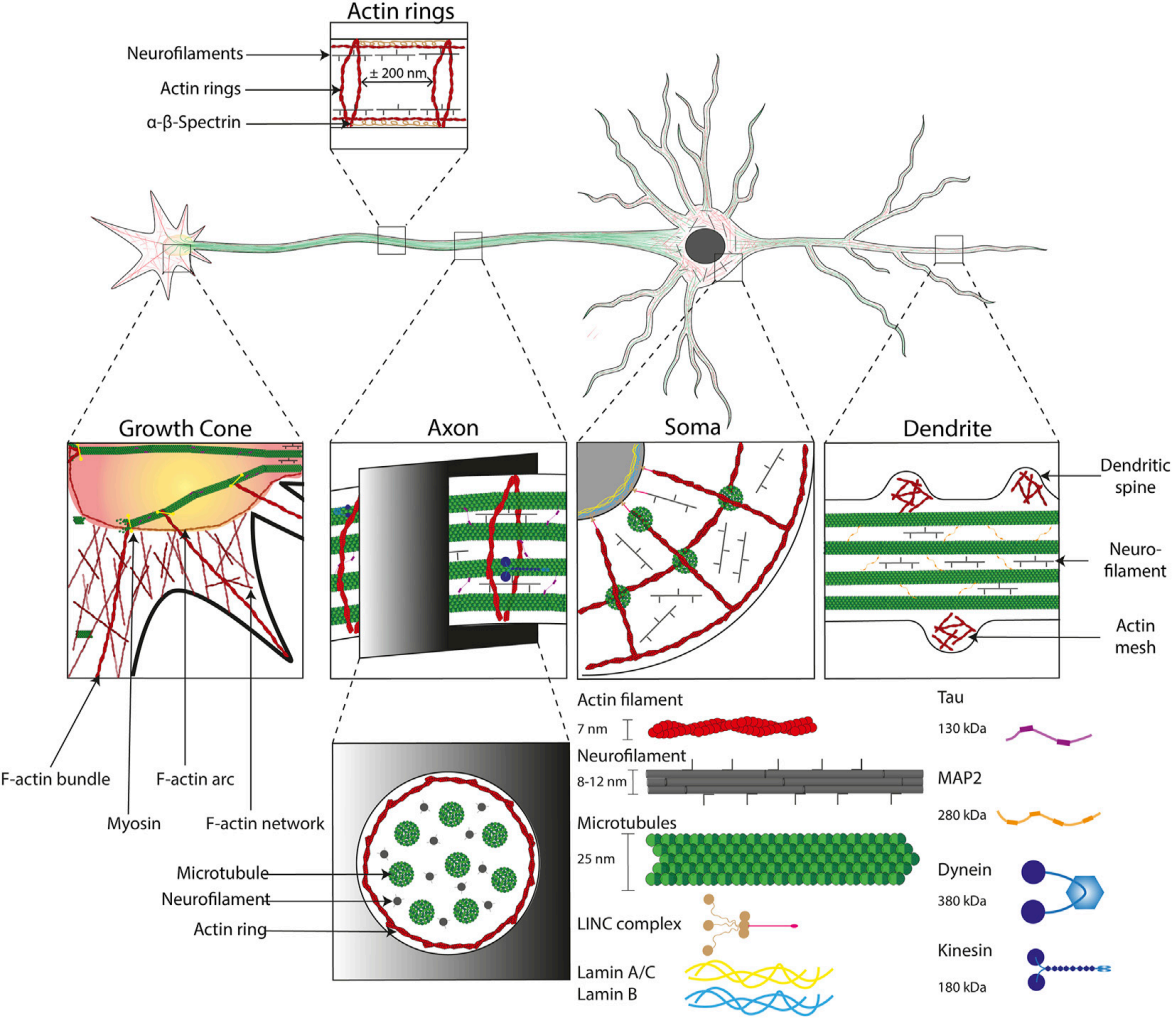
Cytoskeletal organization in neuronal cells. Illustration of the cytoskeletal organization into the four major subcellular compartments of neuronal cells. From left to right: the growth cone, the axon, the cell body (Soma) and the dendrites. The cytoskeleton is composed of i) actin filaments (∼7 nm in diameter, in red) that form specific structures, such as: bundles in growth cones, actin rings (interspace of ∼200 nm) in axons, F-actin arcs in Soma and dendritic spines, ii) neurofilaments (8–12 nm in diameter) which have a supporting role and iii) microtubules (∼25 nm in diameter) stabilized by the Tau protein (130 kDa) in axons and the MAP2 protein (280 kDa) in dendrites. The microtubule network enables the formation of tracks in axons that support axonal transport through molecular motors allowing a retrograde transport (from the growth cone to the Soma) with dynein (380 kDa) and anterograde transport (from the Soma to the Soma) with kinesin (180 kDa). Cytoskeletal components are intimately linked to the nucleus with LINC complex, which is itself anchored to the lamin network (14–30 nm thick) inside the nucleus.

The cytoskeleton of neuronal cells is composed of three main polymers: microtubules (MTs), intermediate or neurofilaments (NFs), and actin filaments or microfilaments (MFs). While they form an actin mesh in the dendritic spines (Star et al., 2002), MFs can be described as highly repeated patterns in the axons known as actin rings spaced of approximatively 180–200 nm and be linked by α-β-spectrin dimers (Xu et al., 2013; Vassilopoulos et al., 2019). Neurofilaments are approximately 10 nm in diameter and are made of heteropolymers, composed of subunits of variable molecular weight as well as internexin or peripherin intermediate filaments (Leterrier et al., 2017). They are in high density in axon from 170/µm^2^ in internodes to 209/µm^2^ in nodes (Reles and Friede, 1991). NFs are heteropolymers forming arms when glial filaments (GFs) are homopolymers without any arms (Pigino et al., 2012). MTs lead to the formation of highly dynamic regions, as well as stable regions, made of α-tubulin and β-tubulin heteropolymer. MTs have a diameter of ∼25 nm and are polar structures, with a fast-growing ‘plus’ end and a slower-growing ‘minus’ end. MTs stabilize the cellular architecture during rest and movement thanks to their ability to bear mechanical loading (Figure 3). Furthermore, they serve as tracks for the movement of mitochondria, lipids, synaptic vesicles, proteins, and other organelles (Ciocanel et al., 2020). Axonal transport is divided into the slow transport of cytoplasmic proteins (including enzymes and cytoskeletal structures such as NFs) and the fast transport of membrane-bounded organelles along microtubules. Fast axonal transport is based on the predominant role of kinesin and dynein with a transport rate of 50–400 mm/day (Cyr and Brady, 1992; Maday et al., 2014). Although dynein and kinesin role has been revealed in fast axonal transport, a new hypothesis have been proposed that consists on a “stop and go” process to explain the slower transport rate by the pausing of molecular cargoes (Brown et al., 2005; Brown and Jung, 2013). This intracellular traffic can be described as anterograde flow (toward the synapses) supported by the kinesin and retrograde flow (toward the Soma) mainly produced by the dynein molecular motors (Guillaud et al., 2020).

Emerging evidence suggests that the nucleus of brain cells must be considered as a key mechanical ingredient. Mechanical properties of the nucleus are mostly related to the chromatin state and the nuclear lamina, which is connected to the cell cytoskeleton by LINC complexes (Corne et al., 2017). The nuclear lamina is an intermediate filament meshwork, composed largely of lamins A and C (A-type lamins) and lamins B1 and B2 (B-type lamins), that is located immediately adjacent to the inner nuclear membrane and provides a structural scaffolding for the cell nucleus. Among the different lamin types, the expression level of A-type lamins mainly determines nuclear stiffness and viscoelastic properties (Swift et al., 2013). Nonetheless, B-type lamins also contribute to nuclear stiffness and stability, and loss of either lamin type results in abnormal nuclear shape and increased nuclear envelope (NE) rupture. However, the level of A-type lamin expression in the human brain is unclear. It was shown recently that cortical glial cells and neurons in the cortex of rat brains both express more lamin C than lamin A (Takamori et al., 2018). Interestingly, astrocytes and oligodendrocytes, both mature glial cells, showed a similar balance of lamins A and C, whereas microglia showed low expression of lamins A and C. Oligodendrocyte progenitor cells showed a weak lamin C immunoreactivity but an intense lamin B1 immunoreactivity. The staining intensity of lamin B2 in all glial cells was found to be relatively weak compared with cortical neurons. These data indicate that glial cells in the adult cerebral cortex showed cell type specific lamin expression patterns (Takamori et al., 2018). However, deregulation of lamin expression can lead to pathologies such as Huntington’s disease and to was shown recently that neurons overexpressing B-type lamin contributes to nuclear dysfunction in Huntington’s disease (Alcalá-Vida et al., 2021). Further studies will be needed to determine the exact role of lamins in the establishment of the mechanical properties of brain tissues. Indeed, the hetero-distribution of the isoforms of lamin can be considered as both static and dynamic biomarkers of mechanophenotype (González-Cruz et al., 2018). Considering that stiff cells express higher ratio of lamin A/C:B than soft cells, it could be interesting to further study lamin expression in brain cells which could be considered as a key factor to the overall brain tissue mechanical properties.

The nucleus is also an interesting organelle for understanding other cellular events such the migration of brain cells. Indeed, migration is a complex cellular process that requires the cytoskeleton to translocate the nucleus through interstitial pores of submicron size. In many situations, dynein and kinesin molecular motors directly interact with the nuclear lamina via the LINC complex and steer directional nuclear movement, while actomyosin contractility and its global flow exert forces to deform and move the nucleus (Figure 2). There is growing evidence that the clutch machinery mediates neuronal migration, which is regulated by extracellular guidance cues. In this context, recent efforts have been made to refine the molecular clutch model that describes the mechanosensing mechanism during axon outgrowth and cell migration (Elosegui-Artola et al., 2018). During such processes, the cytoskeleton is constantly polymerizing, and actomyosin generates contractile forces that push against the membrane. This constant flow of actin is called the “retrograde flow” and is directed from cell edge to nucleus. The motor-clutch was introduced to describe the mechanism that the force imparted to the ECM counteracts myosin contractility, slows down retrograde actin flow, and promotes actin protrusion away from the cell center (Mitchison and Kirschner, 1988). The motor-clutch model predicts how cell migration is affected by the matrix stiffness by considering the number of myosin II molecular motors and clutches to predict the traction force dynamics (Chan and Odde, 2008; Bangasser et al., 2013; Riaz et al., 2016). In the molecular-clutch mechanism, ligand-bound adhesion receptors are mechanically coupled to the actin network to transmit traction forces to the substrate, resulting in a local diminution of the retrograde flow and forward progression. This model was then refined to consider the role of N-cadherin adhesion molecules in the axon outgrowth mechanism. Indeed, it was shown that the growth cone velocity and the mechanical coupling are strongly correlated with N-cadherin receptors and the retrograde actin flow (Bard et al., 2008). To get further insight into this mechanism, experiments using optical tweezers and microneedle were performed to control the motion of microspheres coated with purified recombinant N-cadherin. These experiments have shown a slippage of cadherin-cytoskeletal bonds at low forces and a local actin accumulation with a strengthening of nascent N-cadherin contacts at higher forces (Bard et al., 2008), demonstrating that a molecular clutch between the actin flow and N-cadherin adhesions drives growth cone advance and neurite extension. In addition, motor-clutch models must also include microtubule dynamics and to explain why microtubules-targeting agents (MTAs) can influence cell migration in tumors. Indeed, it was shown that the human glioma sensitivity to stiffness was impaired in MTAs, such as paclitaxel and vinblastine (Prahl et al., 2018). Interestingly, MTAs did not only influence microtubule dynamics but also cell traction forces in an opposite way. Motor-clutch model predictions obtained by computational simulations suggested that MTAs indirectly influence motor-clutch parameters rather than acting directly on tension exerted by the actomyosin network (Prahl et al., 2018). To better identify the clutch molecules involved in neuronal migration and to discriminate from those involved in axonal guidance, recent works have studied the role of Shootin molecules, which are neuronal polarization molecules. Shootin1a was identified as an axonal clutch molecule that accumulates at the leading edge of the axonal growth cone and couples the actin retrograde flow *via* L1-CAM adhesion molecules (Toriyama et al., 2006). Interestingly, a mechanical motor-clutch mechanism based on Shootin1a was recently reported on dendritic spines and highlights the role of synaptic activation in enhancing the actin-adhesion coupling in spines (Kastian et al., 2021). Indeed L1-CAM, laminin, and N-cadherin molecules cooperatively contribute to shootin1a-mediated actin-adhesion coupling to promote robust spine plasticity. In addition to Shootin1a, Shootin1b which is a splicing isoform of Shootin1a, was reported as an important mediator of the mechanical clutch by coupling F-actin retrograde flow at the growth cone with cell adhesions to produce the requisite force for the neuronal movement (Minegishi et al., 2018).

Remarkably, some studies have reported that non-neuronal cells migrating in 3D environments can use a wide range of alternative migration modes that may not involve F-actin-adhesion coupling (Petrie et al., 2014; Stroka et al., 2014), thereby raising the question of whether molecular-clutch models can describe the migration of neuronal cells *in vivo*. To answer this, live *in situ* imaging, super-resolution microscopy and 3D-traction force microscopy were performed on axon growth in 3D microenvironments (Santos et al., 2020). It was shown that neurons grow in an amoeboid mode without the need for adhesions, suggesting that regeneration of adult CNS axons might be facilitated by an amoeboid mode of growth rather than the actomyosin contractive mesenchymal migration mode. This study highlighted the need for a better understanding of the cytomechanics underlying axon growth and to refine molecular-clutch models to 3D microenvironments. Further investigations will require to develop computational and mathematical models at the filament level to consider forces and deformations of individual cytoskeletal components of neuronal cells (Rutkowski and Vavylonis, 2021). Moreover, additional work using mechanobiology assays, such as substrate stretching and optical tweezers, are required to gain quantitative insight into the role of the rate of force application, which has been recently identified as a key component of the mechanosensing mechanism in mouse embryonic fibroblasts (Andreu et al., 2021).

Altogether these findings demonstrate that the nucleus can play an important role in brain tissue mechanics and highlight the importance of the spatial location of brain cells in the establishment of their mechanical properties. To better understand why brain cell mechanics could be modulated by their place of origin, we will describe in the next section the role of the ECM in the modulation of brain cell function.

## ECM Modulates Brain Cell Function

There is growing evidence that the mechanical properties of the cell microenvironment are involved in the normal brain tissue functioning but also in neuropathological situations. Neurons propagate signals through axons and dendrites via complex biochemicals and ionic transfer between extra- and intra-cellular compartments. Action potentials lead to volumetric changes by propagating membrane deformations along the axon (Hill, 1950). Moreover, it was shown that dendritic spines twitch (Crick, 1982) and that rapid actin-mediated contractions occurred after a synaptic activity (Star et al., 2002). These mechanical cues are supported by the cytoskeleton of neuronal cells, which interacts with ECM components via transmembrane integrins (Figure 3).

The brain ECM, which is considered as the softest matrix of the human body, is composed of a variety of proteins that combine to create a complex network of specific biochemical and mechanical properties. The brain ECM is mainly composed of glycosaminoglycans, either bound to proteins, thus forming proteoglycans, or unbound in the form of hyaluronan (Maeda, 2015). The last is a high-molecular-weight protein that acts as a diffusional barrier. Its role is to modulate the diffusion of proteins and nutrients in the extracellular space locally. Consequent to the matrix degradation, hyaluronan fragments are released to the extracellular space where they passively act as pro-inflammatory molecules and give rise to resident immune cells activation, such as microglia (Soria et al., 2020). Brain ECM contains also a small number of fibrous proteins, such as laminins or fibronectin, which have an essential role in cell anchoring (Novak and Kaye, 2000). Although the overall composition of the brain ECM has been mostly determined, the spatial distribution of ECM components is still unclear. Indeed ECM networks in the brain differ in composition, and are spatially distributed (Dauth et al., 2016). The distribution of specific proteins such as aggrecan, brevican, and tenascin-R indicated the presence of large numbers of perineuronal nets (PNNs) in the isocortex, which correlated with clusters of aggrecan (Dauth et al., 2016). This observation suggests that aggrecan in the isocortex is mainly part of PNNs but less abundant in the interstitial matrix, whereas the brevican was observed in the hippocampus at high intensity levels, but not colocalized with PNNs (Dauth et al., 2016). Another important component of the ECM is the large glycoprotein reelin, which is an essential building block of the brain ECM that is secreted by Cajal-Retzius cells in the developing cerebral cortex and hippocampus (Tissir and Goffinet, 2003). Reelin acts as a key regulator of neuronal migration, axonal and dendritic branching, cell aggregation, dendrite formation and synaptic plasticity (Fatemi, 2005; Santana and Marzolo, 2017). It was suggested recently that the migration of multipolar neurons in the developing neocortex follows a multi-step mechanism in which Reelin activates Rap1, Rap1 up-regulates N-cadherin, and N-cadherin is needed to orient cell migration. In addition, it has been observed that Reelin is expressed in several adult neuronal cells, including glutamatergic cerebellar granule neurons and specific GABAergic interneurons of the cerebral cortex and hippocampus (Pesold et al., 1999). Furthermore, accumulative evidence indicates that in the adult brain reelin modulates hippocampal long-term potentiation (LTP) (Herz and Chen, 2006) and synaptic activity (Qiu et al., 2006), promotes hippocampal dendrite and spine development and enhances cognitive ability (Rogers et al., 2011). Dysfunctions in reelin signaling were associated with brain lamination defects such as lissencephaly and with neuropsychiatric diseases as autism, schizophrenia, and depression (Ishii et al., 2016), demonstrating that distinct ECM compositions form specific cellular microenvironments that contribute to brain pathologies (Hemphill et al., 2011).

In addition to the modulation of the biochemical composition of the ECM, modifications of its mechanical properties can affect the spreading, differentiation, migration, or even the epigenetic expression of brain cells (Soria et al., 2020). Using Xenopus retinal ganglion cells (RGCs), it was shown that mechanosensing is critical for axon growth in the developing brain (Koser et al., 2016). Axonal migration was found more persistent on stiff substrates (1 kPa), while it was significantly reduced on softer substrates (0.1 kPa). This result agrees with the presence of a stiffness gradient in the developing brain tissue that should guide the axon growth towards the softer brain tissues. Interestingly, the axonal mechanosensing process was observed to be mediated by piezo1 stretch-activated ion channels (Koser et al., 2016). Aberrant axon growth in response to the softening of brain tissue obtained by manipulating the ECM component, suggesting the reorganization the ECM during the developmental brain can lead to an impaired development. Altogether, these results demonstrate that the local tissue stiffness, which is read out by the piezo1 mechanosensitive ion channels, is critically involved in instructing neuronal growth *in vivo*.

Recent efforts have been made to decipher the role of matrix deformations on glial cells differentiation and more precisely on oligodendrocytes, which are part of the interstitial neuroglia. The main function of oligodendrocytes is the formation of the myelin sheath that wrap the axons of the CNS, whereas this function is assumed by Schwann cells in the peripheral nervous system (PNS) (Marton et al., 2019). While chemical cues are well known to enhance the differentiation oligodendrocytes, recent evidence suggests that biophysical properties of the ECM such as stiffness, topography or strain can also be involved (Jagielska et al., 2012; Shah et al., 2014). For instance, a mechanical strain can stimulate oligodendrocyte differentiation in a ligand-dependent manner, whereas it can inhibit its proliferation through changes of the nuclear shape and global gene expression (Jagielska et al., 2017; Makhija et al., 2018). Modifications of matrix stiffness can be detected by cells that exert traction forces on their substrate through the establishment of focal adhesions (Lampi and Reinhart-King, 2018). In response to stiffness changes, the contractile actomyosin network adapts the cortical tension at the global cell scale acting on the overall tissue stiffening (Clark et al., 2007; Fouchard et al., 2011).

In neurobiology, important efforts have been made to understand how mechanical events can establish physiological cellular functions. For example, it has been demonstrated that matrix stiffness can modulate the formation and activity of cortical neurons *in vitro* (Lantoine et al., 2016)*.* Indeed, the migration of cortical neurons was found to be enhanced on soft substrates, leading to a faster formation of neuronal networks. However, pre-synaptic density, number of action potentials, and miniature synaptic currents were enhanced on stiff substrates (Lantoine et al., 2016). Other works reported that stiff substrates promote neurite outgrowth of cortical and hippocampal neurons (Flanagan et al., 2002; Kostic et al., 2007; Athamneh and Suter, 2015) and enhance neuronal activity (Zhang et al., 2015). Despite these efforts, the mechanotransduction pathways involved in neuronal cell migration and neurite outgrowth are not clear yet. Axonal elongation is mediated by the growth cone, which can be influenced by chemical and physical ECM cues. Forces generated by the growth cone pulling itself are thought to be the motor that pulls the axon along the substrate by actomyosin-mediated contraction (Short et al., 2016).

Mechanical and functional properties of brain tissues are modulated by a set of tunable features. The composition of the ECM can modulate the cell mechanical properties through a spatial reorganization of the cell cytoskeleton, which can in turn modify the mechanical properties of brain tissues. Despite recent advances, how brain cells can sense the physico-chemical changes of their microenvironment and how these changes can lead to a regulation of their functions are still open questions in brain cell mechanotransduction.

## Mechanosensitivity and Mechanosensing

The mechanical properties of the ECM of the CNS have some distinctive and unique features regarding mechanics, structure, and composition that differ substantially from the ECM of other organs and tissues. The process of converting mechanical stimuli into biochemical signals is called mechanotransduction (Martinac and Cox, 2017) and is critical for the function and survival of brain cells. In the following, we will therefore focus on the mechanosensing machinery, which is used sense and interpret microenvironmental biophysical features, highlighting recent findings in neuromechanics. Brain ECM stiffness has been recently studied by observing the activation of astrocytes in response to matrix softening, while matrix stiffening reverted the process (Hu et al., 2021). Interestingly, changes of the brain ECM stiffness were also observed to impact microglia, which is the first line of defense after infection or trauma and is an active actor of synapses remodeling (Yates, 2020). Indeed, changes in matrix stiffness were found to induce a morphological adaptation of microgliocytes. It was reported that soft substrates enhanced microglia polarization and increased their proliferation (Blaschke et al., 2020). Similar to other cell types, microglial cells were observed to migrate towards the stiffer zone of a mechanical gradient (Bollmann et al., 2015). However, few works have reported that neurons migrate toward a softer environment, a process known as negative or inverse durotaxis. This mechanism first observed in the developing embryonic brain of *Xenopus* (Franze et al., 2013; Koser et al., 2016) was also found recently in human glioblastoma cells. Interestingly, inverse durotaxis was not observed to be related to focal adhesion kinase (FAK), extracellular signal-regulated kinase (ERK) or Yes-associated proteins (YAP) signaling. A better understanding of the molecular pathways implicated in the mechanosensing process of brain cells will be substantial for designing new implants or enhancing therapeutic strategies for neurodegenerative pathologies or traumatic brain injuries.

Topotaxis is another process related to ECM fibers that mediates directional cell migration in response to the gradients of the density of extracellular matrix fibers (Park et al., 2016). Studies on hippocampal neurons showed that neuronal growth was random compared to culture on pillared surfaces. They observed the longest neurites lengths on pillars with the smallest inter-pillar space (2 µm) (Dowell-Mesfin et al., 2004). These observations were confirmed by observing that neurons formed longer axons on lines than on holes and smooth surfaces, but independently of their orientation (Fozdar et al., 2010). More precise micropillar arrays fabricated with a laser were able to control the direction of neurite outgrowth of DRGs neurons and Swann cells (Simitzi et al., 2015). However, the underlying mechanosensing mechanisms are not yet elucidated. To better understand how nanotopographical features affect neuronal adhesion, morphology, and neurite outgrowth, a proteomic analysis reported that important proteins were upregulated, while many others were downregulated on surfaces with nanoscale topographical features (Schulte et al., 2016). A myriad of axon-guidance signaling pathways, including synaptogenesis and synaptic regulation, were found to be upregulated (Baranes et al., 2019). Altogether these observations demonstrate the importance of mechanosensing mechanisms in physiological processes of brain cells. The emerging role of substrate topography in brain cell fate allows promising opportunities towards a better understanding of complex developmental processes or the design of new regenerative platforms.

### Mechanosensitivity of Brain Cells

It has been shown that neuronal membranes and membrane channels can be modulated by mechanical stimuli, which affect neuronal activity (Morris, 2011; Tyler, 2012) and indicate that neuronal cells are mechanosensitive cells. Mechanosensing can be described as an active cellular process through which cells detect changes of external forces or mechanical properties of their microenvironment (Chalfie, 2009). However, little is known about the magnitude of forces required for neurons to respond to internal and external mechanical stimuli. It has been shown that forces experienced during a collision, or a shock can lead to diffuse axonal injury (DAI) (Hemphill et al., 2011), membrane poration (Kilinc et al., 2008), and ultimately apoptosis (Serbest et al., 2005, 2006). However, how sub-traumatic forces are sensed by brain cells (Gaub et al., 2020) and transduced in activity changes remain to be described. In this context, identification of novel mechano-gated ion channels and their modulators is essential for understanding mechanosensitivity in neurons and other brain cells.

The first experimental demonstration that mechanical forces could directly activate ion channels was the activation of ionic currents in auditory epithelial cells by Hudspeth and Corey in 1979 (Corey and Hudspeth, 1979). Subsequently, ionic channels in the membrane of tissue-cultured pectoral muscle were observed to be activated by membrane stretch (Guharay and Sachs, 1984). Ion channels that are directly (i.e., sub-millisecond range) activated by a mechanical stimulation are classified as mechanically activated channels. Among the 15 members of the K_2P_ channel family, TREK1 (also known as potassium channel subfamily K member 2) was the first identified mechanically activated mammalian ion channel. Subsequently, TREK2 (also known as potassium channel subfamily K member 10) and TRAAK (also known as potassium channel subfamily K member 4) were discovered (Maingret et al., 1999). K_2P_ activation suppresses neuronal excitability in the sensory system by hyperpolarizing the membrane potential, whereas asymmetric tension induced by membrane curvature is sufficient to activate K_2P_ channels (Ranade et al., 2015). Transient receptor potential (TRP) channels could modulate ion entry driving forces and Ca^2+^ and Mg^2+^ transport machinery in the plasma membrane (Clapham, 2003). Among them, TRPV4 was reported insensitive to mechanical indentation and membrane stretch, but to open in response to elastomeric pillar array-mediated membrane stretch (Rocio Servin-Vences et al., 2017). In addition, TMEM150C/Tentonin3 was proposed to act as an ion channel mediating slowly inactivating mechano-evoked current in proprioceptive neurons in mouse DRG neurons but heterologous expression of TMEM150C fails to generate MA currents in cells with genomic ablation of the PIEZO1 gene (Hong et al., 2016; Dubin et al., 2017). By cloning TMEM150C from the trigeminal neurons of the tactile-foraging domestic duck it was shown that TMEM150C must be considered as a general regulator of mechano-gated ion channels from different classes (Anderson et al., 2018).

Piezo channels family is formed by two genes: Piezo1 and Piezo2 that share half amino acid composition when the protein is expressed in vertebrates, and are the largest known pore-forming multimeric ion channels, with ∼2,500 amino acids in each subunit (Murthy et al., 2017). Mechanical activation of the Piezo channel results in influx of Na^2+^ and Ca^2+^, which can lead to the propagation of electrical signals and initiate intracellular secondary messenger pathways. PIEZO1 is expressed mainly in non-neuronal cells, whereas PIEZO2 is expressed in sensory neurons and specialized mechanosensory structures. PIEZO1 and PIEZO2 were found to be active players in mechanically-activated currents in Neuro2A and DRG neurons (Coste et al., 2010). Using Yoda1 and Capsaicin agonists, longer mechanical hyperalgesia was observed *in vivo* with the activation of Piezo1 rather than TRPV1, suggesting that Piezo1 is a promising candidate for mechano-nociception (Wang et al., 2019). Piezo1 has been observed to adopt an activated state after axonal injury and during axonal regeneration where chemical activation of Piezo1 by Yoda1 agonist has shown slight inhibition of axonal regeneration via the CamKII-Nos-PKG pathway (Song et al., 2019). In addition, the upregulation of astrocytic Piezo1 may dampen neuroinflammation (Velasco-Estevez et al., 2020). Indeed, activating Piezo1 channels can inhibit the release of cytokines and chemokines, such as IL-1β, TNFα, and fractalkine (CX3 CL1) in activated astrocyte cultures. Furthermore, LPS-stimulated astrocytes exposed to Yoda1 (Piezo1 channel activation) versus GsMTx4 (Piezo1 channel inhibition) indicated that Piezo1 channel activation in reactive astrocytes decreases their migration speed (Velasco-Estevez et al., 2020), suggesting a key role of PIEZO1 in astrocyte functioning. Force from lipid (FFL) models have been used to explain that forces arising from actin-mediated contractility and within the lipid bilayer act synergistically to regulate PIEZO1 activation (Bavi et al., 2019). The FFL gating paradigm implies that mechanical force activates MS channels through the lipid bilayer alone with no requirement for other cellular components (Ridone et al., 2019).

Despite the recent surge in Piezo-dependent mechanotransduction research, several aspects of Piezo channel architecture and physiology are still unknown (Murthy et al., 2017) and the role of Piezo channels in brain functioning remains elusive. Intracellular mechanobiology was largely thought to be mediated by transmembrane proteins such as integrins, but the emerging role of the PIEZO1 ion channel in traction force sensing can potentially remodel this concept. Furthermore, we envision that the identification of phenotypes associated with human brain mutations in PIEZO channels will have a significant impact on our understanding of the key role of mechanotransduction-based processes.

### Integrin-Mediated Mechanosensing

Brain cells interact with the surrounding matrix by using transmembrane integrins, which consist of a 24 heterodimers transmembrane protein family composed of 18 α- and 8 β-subunit (Moreno-Layseca et al., 2019). These integrin heterodimers are not found uniformly in the brain and do not bind the same ECM proteins (Wu and Reddy, 2012). Integrin activation can either be induced by integrin ligand binding itself (outside-in signaling) or intracellular cascades (inside-out signaling) (Mohammed et al., 2019). Interestingly, activation of integrin can also be triggered by a change in membrane tension resulting from mechanical forces or by cytoskeletal forces generated by the actomyosin complex linked to integrins *via* adaptors proteins (Gingras and Ginsberg, 2020). The integrin-ECM has been described as a catch-bond which significates that receptor-ligand liaison strengthens under increasing forces until a threshold (Kong et al., 2009). Cell-matrix adhesion is therefore ruled by the nature of integrins and the stability of the binding. Interestingly, the binding stability has been observed to depend on the level of applied forces which influence in return the integrin clustering (Gauthier and Roca-Cusachs, 2018). This ligand-binding mechanism provides mechanical properties sensitivity for brain cells which means that they can adapt to the mechanical environment such as stiffness and topography. It was shown that talin binds the β-integrin subunit (Shattil et al., 2010; Kim et al., 2012) which destabilizes the interaction with the α-subunit, whereas kindlin seems to act as an indirect activator of integrins by cooperation with talin (Theodosiou et al., 2016).

A large number of studies indicated that soft substrates promote neurite outgrowth for a variety of neuronal cell types (Balgude, 2001; Teixeira et al., 2009; Franze et al., 2013; Kerstein et al., 2015; Lantoine et al., 2016; Mosley et al., 2017). However, other studies reported conflicting results about the sensitivity of cortical (Zhang et al., 2015) and hippocampal (Koch et al., 2012) neuronal cells towards matrix stiffness, suggesting that mechanosensitivity can vary between different neuronal cell types. Interestingly, the intrinsic electrical properties of neurons were observed to change in response to the modifications of the mechanical environment (Lantoine et al., 2016; Wen et al., 2018). Mouse hippocampal neurons cultured on stiff substrates displayed enhanced voltage-gated Ca^2+^ channel currents compared to neurons cultured on soft substrates (Wen et al., 2018). In addition, matrix stiffness can alter mechanically gated ion channels, such as Piezo1, conduct calcium and sodium ions (Coste et al., 2012). In addition to the study of the matrix stiffness, further works are needed to understand whether viscoelastic properties of the matrix (Chaudhuri et al., 2020) can modulate the membrane potential and tune the electrophysiological properties of neurons. Indeed, emerging evidence suggests a time dependence of cellular mechanosensing and the influence of viscous dissipation of the ECM on cell phenotype (Chaudhuri et al., 2016; Janmey et al., 2020). For instance, if this hypothesis is valid, altered tissue mechanics may contribute to calcium dysregulations in the aging or neurodegenerative brains.

## Mechanobiology in Brain and Nerve Diseases

In addition to the important role of biophysical cues in neurophysiological processes, external mechanical forces and changes in the physico-chemical properties of the ECM were also observed to be involved in the development of brain and nerve pathologies. We will discuss in this section how perturbation of the cell microenvironment and modification of the mechanical properties of brain and nerve tissues can contribute to the emergence of specific diseases by focusing on three specific examples: traumatic brain injury, neurodegenerative diseases, and neuroblastoma.

### Traumatic Brain Injury

**FIGURE 4:**
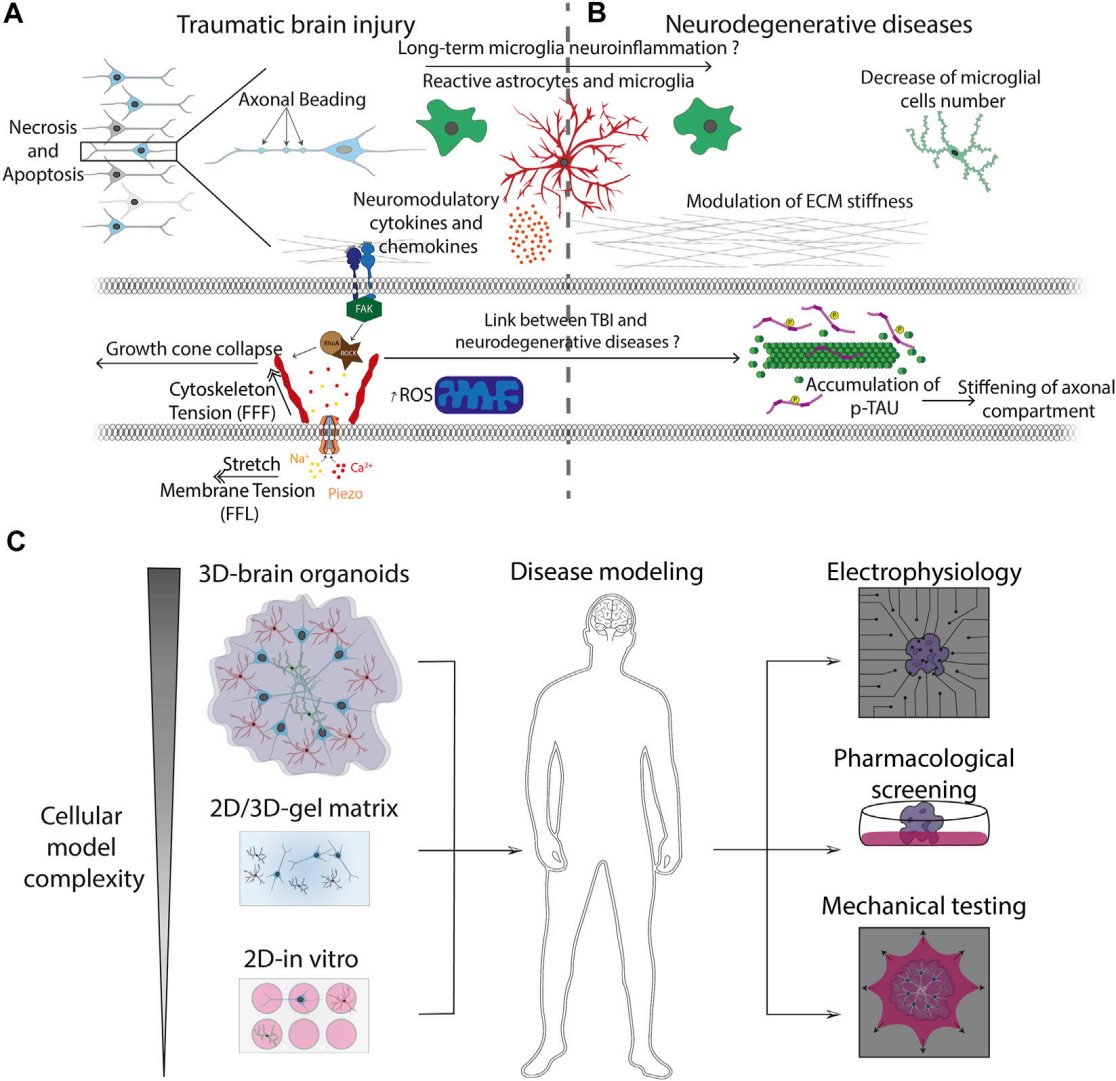
Pathophysiological processes and advanced *in vitro* models **(A)** Mechanically-induced diseases, such as TBI, leads to axonal beading and activation of glial cells. Reactive astrocytes and microglia trigger the release of cytokines and neuromodulatory chemokines that are involved into the long-term neuroinflammation process observed in neurodegenerative diseases. Injury forces applied to neuronal and glial cells are transmitted from the ECM to the cytoskeleton through transmembrane integrins. Focal adhesion kinase (FAK) is a cytoplasmic tyrosine kinase that plays critical roles in integrin-mediated signal transduction and can trigger the activation of the Rho-ROCK pathways, leading to rearrangement of actin filaments and ultimately to the axonal growth cone collapse. **(B)** Cytoskeletal changes are related to the formation of protein aggregates such as phosphorylated the neuronal microtubule Tau protein, which is a hallmark of many neurodegenerative diseases. This process can be followed by a stiffening of the axonal compartment and major modifications of the ECM mechanical properties. **(C)** Advanced bioengineered systems allow to increase the complexity of conventional 2D culture models to obtain physiologically relevant 3D pluricellular models such as brain organoids. The 3D culture models can be combined with mechanical assays and new electrophysiological methods such as microelectrode arrays (MEA) for investigating brain development, neurodegenerative diseases and to improve the screening of pharmacological drug candidates.

Traumatic brain injury (TBI) is a nondegenerative and noncongenitally insult to the brain arising from an external mechanical shock, which can lead to permanent or temporary impairment of cognitive, physical, and psychosocial functions (Sandsmark and Diaz-Arrastia, 2021). TBI can be associated with a diminished or altered state of consciousness and its molecular mechanisms have been extensively studied during the last few decades using innovative *in vitro* assays (Puntambekar et al., 2018). TBI can be separated between primary and secondary injuries. Primary injuries are the direct mechanical consequences of the traumatic event occurring on the brain. It can be described as disruption of cortical parenchyma, axotomy, and massive cellular death (Kaur and Sharma, 2018). The secondary injury is more complex and is triggered by glutamate excitotoxicity (Luo et al., 2019) along with inflammation neuromodulator (Simon et al., 2017), astrogliosis (Burda and Sofroniew, 2014; Burda et al., 2016), and ion dysregulation (Weber, 2012). Primary injury can be described as contusion (Ct) and pericontusion (PCt) (Harish et al., 2015). Ct is the center of TBI where occurs hemorrhage, shrunken neurons, and inflammatory changes. PCt is more the seat of oedema, axonal loss, and dystrophic changes in neurons and astrocytes and where microglial activation occurs. DAI and neurotoxicity, which are key players of the secondary injury, are associated with the dysregulation of glutamate transmission and the release of proteins in extracellular compartments, leading to massive tissue necrosis and apoptosis (Akamatsu and Hanafy, 2020). In addition, axonal mitochondria can be injured, leading to massive formation of reactive oxygen species (Kumar Sahel et al., 2019). Furthermore, accumulative evidence underlines the role of the immune system in microglial activation and astrogliosis in response to inflammation signals in the CNS (Karve et al., 2016). TBI is also implicated in memory loss due to dysregulation of the dopaminergic pathway (Chen et al., 2017). All these features are characteristic of the secondary injury that progresses over time after the traumatic event (Figure 4A).

Among these biochemical events, accumulative evidence suggests a key role of the impact of mechanical forces on tissue remodeling and cellular adaptation. Loading forces can be applied on brain tissues directly (i.e., physical insult) or indirectly (i.e., acceleration-deceleration forces). Although the amount of loading forces applied to the brain is a critical factor, the nature and direction of loading are also important parameters. Indeed, longitudinal, transversal, or rotational stresses can lead to different degrees of severity (Ivancevic, 2009). In addition, TBI can result from a direct impact caused by contact between an object of high energy or from the rapid movement of the head in the space characterized by fast acceleration and deceleration phases (Brazinova et al., 2016). Inertial forces apply longitudinal tension and compression to brain tissues and can cause shear stress when the acceleration is not longitudinal to the axis of the head and neck (Blennow et al., 2012). The mechanical loading of brain tissues often results in focal injuries (skull fracture, contusion, or hemorrhage) and/or diffuse injuries such as diffuse vascular injury, diffuse brain swelling, and DAI.

In response to intense stretching forces, axons can break and neurons can go under cell-autonomous death pathways like Wellerian degeneration (Kanamori et al., 2012). Distal axotomy can also have an influence on injured neurons by enhancing retrograde presynaptic excitability via transsynaptic signaling (Nagendran et al., 2017). Secondary axotomy can be triggered by a stretch of at least 10%, occuring in 100 ms or less (Di Pietro et al., 2013). During the secondary injury, forces are translated to the cytoskeleton by the focal adhesions and detected by mechanosensory proteins inducing intracellular signal pathways. By using stretch assays and optical magnetic tweezers, it was found that ECM-integrin specific interaction activates the RhoA-ROCK signaling pathways (Hemphill et al., 2011), which is known to be implicated in growth cone collapse, therefore inhibiting the growth and repair of axons (Dupraz et al., 2019). When loading exceeds the mechanical limit of the cell, cytoskeletal components can break leading for instance to the fracture of microtubules and compaction of neurofilaments (Chen et al., 1999). Axonal transport is therefore impaired and proteins such as βAPP and phosphorylated Tau protein are accumulated in “retractation bulbs” also called “axonal beading” (Tang-Schomer et al., 2012). We can also witness a rapid and important intake of calcium which can alter the permeability of mitochondrial membrane through activation of calcineurin and therefore activate caspase-mediated apoptosis (Mu et al., 2015). Furthermore, mechanical activation of glial cells has an important role in the outcome of TBI. It was shown in injured neocortex and spinal cord injury that brain tissues altered their mechanical properties and softened, suggesting a soft mechanical signature of glial scars following brain injury (Moeendarbary et al., 2017). The release of cytokines and chemokines triggers the loss of presynaptic vesicles in cortical neuronal networks and modulates the balance between TNFR1 and TNFR2 receptors (Lantoine et al., 2021).

In addition to immediate complications and the development of a neuroinflammatory context, TBI involves not only short- but also long-term consequences, including an increased risk for patients to develop neurodegenerative disorders such as Alzheimer’s disease, chronic traumatic encephalopathy, and amyotrophic lateral sclerosis in later stages of life (Gupta and Sen, 2016). A better consideration of mechanobiological aspects of TBI is critical to move the field forward. Indeed, most of the current injury models are not representative of human injury and thus fail to replicate mechanisms of primary and secondary damages. The development of advanced *in vitro* models that integrate mechanobiology assays is critical to improve our understanding of the cellular response to neurotrauma and to address current open questions, such as determining the molecular pathway involved in stress propagation through brain tissues or establishing effective pharmacological treatments that can be clinically translated.

### Neurodegenerative Diseases

Aging is the main risk factor for humans to develop neurodegenerative diseases, which are defined by progressive degeneration of the structure and function of the central or peripheral nervous system. Neurodegenerative diseases are a group of pathologies, which have cellular and sub-cellular similitudes with programmed cell death, such as protein accumulation within the neurons (Rubinsztein, 2006).

Accumulation of hyaluronic acid, one of the constituents of the ECM, in the dentate gyrus of the hippocampus is correlated with a reduced number of dendritic spines but also with spatial memory impairment (Yoshino et al., 2017, 2018). In addition the accumulation of hyaluronic acid in the cortex and cerebellum of aged mice, suggests that remodeling of the brain ECM with time is not suitable for synaptic plasticity (Foscarin et al., 2017; Reed et al., 2018). Accumulative evidence shows a positive correlation between increasing amyloid load and reduced brain stiffness in mild cognitive impairment (Murphy et al., 2016) and magnetic resonance elastography (MRE) images of Alzheimer patients show a decrease in brain stiffness compared to healthy controls (Murphy et al., 2016; Hiscox et al., 2020).

In addition to ECM changes, many neurodegenerative diseases were associated with important changes in cell composition and cell mechanics. The decrease in the neuron-to-glial cell ratio could contribute to the overall softening of AD brains, at least on the macroscale of MRE elastograms (Figure 4B). Furthermore, several MRE studies revealed a continuous softening of the human brain tissues with age from ∼4 to ∼2 kPa (Sack et al., 2011). Increasing age is the single greatest risk factor for the development of AD (Hou et al., 2019) that is associated with the loss of neurons and synapses in the cerebral cortex and certain subcortical structures (e.g., hippocampus). The loss of neurons results in a volume decrease of different regions of the brain, including the temporal lobe, parietal lobe, and parts of the frontal cortex and cingulate gyrus (Wenk, 2003). Alteration of the viscoelastic properties of the brain ECM was observed occurring in AD (Murphy et al., 2011; Hiscox et al., 2020), with specific differences according to the different brain regions. For instance, association cortices (frontal with 2.65 versus 2.47 kPa and temporal with 2.69 versus 2.58 kPa) showed a significant softening, whereas other region remained unaffected (occipital, sensory/motor, deep gray and white matter, and the cerebellum) (Murphy et al., 2016).

There is now accumulative evidence linking TBI to dementia (Fann et al., 2018; Gardner et al., 2018) and neurodegenerative diseases (DeKosky and Asken, 2017; Brett et al., 2021) that involves the gradual and chronic hyperphosphorylation, misfolding, and missorting of the microtubule-associated protein, tau. When tau is phosphorylated, it detaches from microtubules at the axon initial segment and breaks down the barrier that normally prevents retrograde flow of axonal tau (Hatch et al., 2017). This may cause the neuron to become stiffer due to the accumulation of tau in the somatodendritic compartment (Hagestedt et al., 1989; Zempel et al., 2017). Therefore, hyperphosphorylation of tau causes intrinsic mechanical disturbances in damaged neurons. Because axonal transport is dependent on a normal functioning cytoskeleton, marked tau-associated changes in neuron stiffness may be a novel biomarker of neurodegenerative disease associated with a severe breakdown in axonal transport machinery (Millecamps and Julien, 2013).

### Neuroblastoma

In addition to be involved in CNS diseases, mechanobiology was also found to be a key parameter of other nerve diseases that affect predominantly the peripheral nervous system. Human neuroblastoma (NB) is the most common extracranial solid tumor occurring during infancy (Allen-Rhoades et al., 2018). NB is a neurodevelopmental disorder that can be viewed as resulting from the failure of neural crest differentiation (Ratner et al., 2016). This sympathetic nervous system tumor displays several unique features: the early age of onset, high incidence of metastatic cases at diagnosis, and proneness for spontaneous regression of tumors (Matthay et al., 2016). The origin of NB remains unknown, but accumulative evidence suggests that neural crest cell-derived neuroblasts may be one cell of origin and amplification of the oncogenic transcription factor MYCN was found as the main characteristic of a subset of human NBs (Ratner et al., 2016).

The migratory and aggressiveness potential of NB cells appear to be intrinsically related to their stem cell-like characteristics, such as self-renewal and migratory potential. Consistently, I-type NB cells in tumors present the highest level of progress and the highest malignant potential *in vitro* (Matthay et al., 2016). It was observed recently that a lack of lamin A/C could predispose cells towards stem-like phenotype (Nardella et al., 2015), which is suggested to be involved in therapy failure. This is particularly interesting considering that lamin A/C expression is known to scale with the differentiation state (Swift et al., 2013) and that low lamin A/C level correlates with softer nuclei, which is essential for fast amoeboid migration (Heo et al., 2020). Interestingly, clinical behavior of NB is correlated with high-level amplification of the MYCN oncogene, whose expression was found to inversely relate to lamin A/C expression in tumor-initiating cells (Nardella et al., 2015).

Upon differentiation, the elastic modulus of human-derived NB was observed to increase significantly (approximately 3-fold increase) (Zhao et al., 2015) and to scale with the cell differentiation state. This mechanism is not well understood and it has been suggested that it could originate from an increase in the heterochromatin after differentiation (Bergqvist et al., 2020) or due to structural modifications in the actomyosin cortex after differentiation. Several studies demonstrated that the degree of tubulin polymerization can be four to five times higher in differentiated NB cells possessing microtubule-filled neurites (Van de Water and Olmsted, 1980; Olmsted and Lyon, 1981). Interestingly, the average young’s modulus of NB was shown to increase from 5 to 10 kPa after the induction of neurodegeneration. This cell stiffening was correlated to the activation of the RhoA signaling pathway and induced a consequent increase in contractile forces within the cytoskeleton (Fang et al., 2014). It is likely to assume that elastic moduli of human NB cells can significantly vary during the disease progression and these variations could be correlated with the different malignant potential in NB cell types, as they are associated with different differentiation states (Mescola et al., 2012). Further mechanical experiments need to be conducted to better determine the mechanical properties of NB cells according to their metastatic potential. This could help to understand their migratory potential as they might be more deformable during migration and may constitute a basis for sorting according to metastatic potentials. In addition, novel 3D *in vitro* platforms that efficiently sustain patient-derived tumor cell growth are required for assessing drug-specific responses and performing more robust pre-clinical testing (Corallo et al., 2020).

## Brain-Tissue-Like Biomaterials

One of the major limitations in the study of brain and nerve diseases is the lack of *in vitro* systems that faithfully recapitulates the complexity and delicacy of human brain and nerve tissues. A better understanding of the modulation of brain and nerve function in response to physico-chemical changes of the surrounding matrix or external forces requires to create novel biomaterials with similar mechanical and biochemical properties to the native tissues. Indeed, recapitulating *in vitro* the complex microenvironment of neuron and glial cells is crucial to study their cellular responses to chemical signals, to develop new pharmacological candidates, and to expand cells for therapeutic applications.

Due to the softness and a large amount of water in brain tissues, hydrogels are promising candidates and offer the possibility of delivering drugs in a sustained release manner (Appel et al., 2012; Mol et al., 2019). Hydrogels are water-swollen biomacromolecules that possess up to 90% of water, while exhibiting the physical properties of elastic solids. The high-water content allows the diffusion of biomolecules secreted by cells. Hydrogels are 3D networks that are promising tools for brain tissue regeneration due to their tunable physico-chemical properties. Conventional biomaterials such as autologous bones and titanium meshes are rather stiff, with young’s modulus values ranging from dozen of kPa up to GPa and have poor stretchability. Hydrogel scaffolds can overcome these drawbacks and adapt to intracranial pressure changes and degrade after few weeks (Che et al., 2019). In the past few years, injections of hydrogel directly into the brain have been used in the treatment of nervous system damage diseases (kun Zhang et al., 2018; Sun et al., 2020). Injectable peptide hydrogels in a zebrafish model enable angiogenesis that promotes new blood vessel growth and neurogenesis by increasing neural growth both *in vitro* and *in vivo* (Wang et al., 2017). An injectable self-assembling peptide-based hydrogel that mimics a vascular endothelial growth factor was used recently to create a regenerative microenvironment for neovascularization at the injured brain tissues (Ma et al., 2020). Indeed, brain damage following significant TBI commonly results in extensive tissue loss and long-term disability. However, there are currently no clinical treatments to prevent the resulting cognitive impairments or tissue loss. To address this limitation, chondroitin sulfate glycosaminoglycan (CS-SAG) matrices were developed to act as a scaffolding for transplanted stem cells, which are capable of repairing damaged tissue in a more natural healing environment (Betancur et al., 2017). Injection of CS-SAG matrices into rats with TBI has led to a significantly enhanced retention of neural stem cells. In addition, CS-SAG matrices were implanted into rats with severe TBI, who after 20 weeks exhibited enhanced cell repair and improvement of motor function (Latchoumane et al., 2021), providing evidence that the hydrogel protects against brain tissue loss, but also actively regenerates functional neurons at the lesion cavity after a TBI.

In addition to these advanced culture matrices that can recapitulate important characteristics of the native microenvironment of neuron and glial cells, the emergence of 3D organoids has attracted great attention in fundamental neurosciences and regenerative medicine. Brain organoids are a type of organoids that tend to reproduce specific brain structures which are characteristic of different human brain regions such as the hippocampus (Sakaguchi et al., 2015), hypothalamus (Qian et al., 2016), or the cerebellum (Muguruma et al., 2015). Interestingly, microfluidic chips have simplified the manufacturing process of brain organoids and micro-pillar array devices are used for *in situ* formations of plentiful brain organoids (Zhu et al., 2017). Brain organoids are now considered as a versatile tool for screening therapeutic compounds for neurodegenerative diseases that allow for instance to observe the aggregation of amyloid-beta and tau pathology (Kim et al., 2019). Recently, human cerebellar organoids were shown to be an effective model to explore *in vitro* the role of genetic mechanisms in glioma patients (Ballabio et al., 2020) and are expected to be relevant models for mechanical testing and electrophysiological studies (Figure 4C).

## Future Perspectives and Challenges in Mechanobiology of Brain Tissues

Over the last decade, neuroscience studies have extensively used electrophysiology, molecular biology, biochemistry, and genetics. At the same time, the emergence of cell mechanobiology gained many other fields and the role of mechanical forces in mediating neuronal processes remained unexplored. Accumulative evidence shows that the brain is a mechanically sensitive organ and that its structure and functioning can be regulated by external and internal forces (Tallinen et al., 2016), for instance during neural migration (Abuwarda and Pathak, 2020) and formation and pruning of synapses (Sakai, 2020). Studying the consequences of endogenous forces exerted on glial cells will help to understand how mechanical injuries to the brain can be lead to the emergence of neurodegenerative diseases via the establishment of a neuroinflammatory context (Kokiko-Cochran and Godbout, 2018).

Among mechanical stimuli, the physico-chemical properties of the matrix stiffness contribute significantly to the neuronal cell fate. Interestingly, changes in tissue mechanics contribute to age-related cognitive decline and neurodegenerative states. Future works are required to understand how changes in composition and mechanical properties of the matrix are linked with neurophysiology and cognition. In this context, a large effort is required to understand how neuronal and glial cell mechanics and brain tissue mechanobiology are intimately connected. An emerging theme concerns the role of the physical changes in the tumor microenvironment that can activate signaling pathways leading to ECM remodeling, which can in turn enhance pro-tumorigenic mechanosignaling. A closer examination of the mechanoreciprocity circuit is required to understand the role of ECM changes in therapy resistance, poor prognosis and to identify new therapeutic targets.

Conventional 2D cell cultures have allowed to identify important cellular signaling pathways, determine potential drug targets and establish the design of drug candidates for brain pathologies. However, cell culture performed in plastic flasks or flat Petri dishes also have many limitations, such as the disturbance of interactions between the cellular and extracellular environments, changes in cell morphology, culture in homogeneous media or limited cell interactions that mainly depend on cell distribution and proximity. To address these drawbacks and facilitate the translation to the clinic, many research efforts have been dedicated to design compartmentalized microsystems (Hur et al., 2011; Bhatia and Ingber, 2014; De Vitis et al., 2021). Microengineering culture platforms represent a promising technology for the study of complex spatiotemporal signals, cell dynamic and pharmacologic response with unprecedented levels of control (Polini et al., 2019). Through the years, microengineering platforms had experienced few variations and are now considered as a powerful tool to study the structure-function relationship and the complex communication between the different brain cell populations in standardized conditions. Promising models combine engineering methods with stem cells, such as embryonic stem cells (ESCs) or human-induced pluripotent stem cells (hiPSCs), to create patterned brain organoids (Geraili et al., 2018; Smith et al., 2020) that will represent a unique opportunity for biopharmaceuticals, cell-based therapies, and personalized medicine.

Despite significant advances in the development of brain organoid cultures (Strzyz, 2021), there are still some issues that need to be addressed such as the harvest difficulty after long cultures or the variability in size and composition (Shou et al., 2020; Passaro and Stice, 2021). Recently, cerebral organoids derived from human, gorilla and chimpanzee cells were used to study developmental mechanisms driving evolutionary brain expansion (Benito-Kwiecinski et al., 2021). Early cell shape transition of the neuroepithelial cells was associated with apical constrictions and elongation and correlated with a slowing down of the cell cycle and DNA transcription. This phenomenon happened at earlier stage in ape-derived organoids than in human-derived organoids, assessing the fundamental mechanisms driven evolutionary expansion of the human forebrain.

We envision that the next generation of microengineered platforms will integrate mechanical assays and advanced synthetic matrices with human brain organoids (Sato et al., 2009; Takasato et al., 2015; Turco et al., 2017; Fujii et al., 2018; Pérez-González et al., 2021) to form an effective preclinical platform that can test and guide personalized treatment in a reproducible and predictable manner. These advanced engineered platforms will bridge the gap between 2D human cell cultures and non-human animal models. Moreover, by integrating relevant surrounding matrices and mechanical constraints they will open the door for a variety of studies including development and disease modeling and high-throughput screening. Finally, a significant effort will be required to overcome the limitations posed by the three-dimensionality of brain organoids in order to develop robust functional analysis methods, such new as electrophysiological approaches (Passaro and Stice, 2021).
